# Biochemical characterization of L-asparaginase isoforms from *Rhizobium etli*—the boosting effect of zinc

**DOI:** 10.3389/fchem.2024.1373312

**Published:** 2024-02-22

**Authors:** Joanna Sliwiak, Paulina Worsztynowicz, Kinga Pokrywka, Joanna I. Loch, Marta Grzechowiak, Mariusz Jaskolski

**Affiliations:** ^1^ Institute of Bioorganic Chemistry, Polish Academy of Sciences, Poznan, Poland; ^2^ Department of Crystal Chemistry and Crystal Physics, Faculty of Chemistry, Jagiellonian University, Krakow, Poland; ^3^ Department of Crystallography, Faculty of Chemistry, Adam Mickiewicz University, Poznan, Poland

**Keywords:** Class 3 L-asparaginase, ReAV, enzymatic mechanism, amidohydrolase, enzyme kinetics, *Rhizobium etli*, zinc metalloprotein, ReAIV

## Abstract

L-Asparaginases, divided into three structural Classes, catalyze the hydrolysis of L-asparagine to L-aspartic acid and ammonia. The members of Class 3, ReAIV and ReAV, encoded in the genome of the nitrogen fixing *Rhizobium etli*, have the same fold, active site, and quaternary structure, despite low sequence identity. In the present work we examined the biochemical consequences of this difference. ReAIV is almost twice as efficient as ReAV in asparagine hydrolysis at 37°C, with the kinetic K_M_, k_cat_ parameters (measured in optimal buffering agent) of 1.5 mM, 770 s^-1^ and 2.1 mM, 603 s^-1^, respectively. The activity of ReAIV has a temperature optimum at 45°C–55°C, whereas the activity of ReAV, after reaching its optimum at 37°C, decreases dramatically at 45°C. The activity of both isoforms is boosted by 32 or 56%, by low and optimal concentration of zinc, which is bound three times more strongly by ReAIV then by ReAV, as reflected by the K_D_ values of 1.2 and 3.3 μM, respectively. We also demonstrate that perturbation of zinc binding by Lys→Ala point mutagenesis drastically decreases the enzyme activity but also changes the mode of response to zinc. We also examined the impact of different divalent cations on the activity, kinetics, and stability of both isoforms. It appeared that Ni^2+^, Cu^2+^, Hg^2+^, and Cd^2+^ have the potential to inhibit both isoforms in the following order (from the strongest to weakest inhibitors) Hg^2+^ > Cu^2+^ > Cd^2+^ > Ni^2+^. ReAIV is more sensitive to Cu^2+^ and Cd^2+^, while ReAV is more sensitive to Hg^2+^ and Ni^2+^, as revealed by IC50 values, melting scans, and influence on substrate specificity. Low concentration of Cd^2+^ improves substrate specificity of both isoforms, suggesting its role in substrate recognition. The same observation was made for Hg^2+^ in the case of ReAIV. The activity of the ReAV isoform is less sensitive to Cl^−^ anions, as reflected by the IC50 value for NaCl, which is eightfold higher for ReAV relative to ReAIV. The uncovered complementary properties of the two isoforms help us better understand the inducibility of the ReAV enzyme.

## Highlights

1) The two L-asparaginases, ReAIV and ReAV, encoded by the nitrogen fixing *Rhizobium etli*, have the same fold, active site, and quaternary structure, despite low sequence identity.
2) ReAIV is about twice as efficient as ReAV in asparagine hydrolysis at 37°C. The activity of both isoforms is boosted by 32% and 56%, respectively, by low concentration of Zn^2+^, which is bound three times more strongly by ReAIV relative to ReAV, as reflected by the K_D_ values.
3) ReAIV has a temperature optimum at 45°C–55°C, whereas the activity of ReAV, after reaching its optimum at 37°C, decreases dramatically at 45°C.
4) The divalent cations Hg^2+^, Cu^2+^, Ni^2+^ and Cd^2+^ have the potential to inhibit both isoforms in the following order (from strongest to weakest inhibitory effect) Hg^2+^ > Cu^2+^ > Cd^2+^> Ni^2+^. However ReAV is more resistant to Cu^2+^ and Cd^2+^, and ReAIV to Hg^2+^ and Ni^2+^. Low concentration of Cd^2+^ improves the substrate specificity of ReAV and ReAIV, suggesting its role in substrate recognition. The same observation was made for Hg^2+^ in case of ReAIV.
5) The activity of the ReAV isoform is less sensitive to Cl^−^ anions, as reflected by the IC50 value for NaCl, which is eightfold higher for ReAV relative to ReAIV.

## 1 Introduction

L-Asparaginases are enzymes catalyzing the hydrolysis of L-asparagine to L-aspartic acid and ammonia. L-Asparaginases can be divided into three structural Classes (Borek and Jaskolski, 2001; da Silva et al., 2022; Loch and Jaskolski, 2021). Enzymes from Class 1 are usually tetramers, formed as a looser dimer of two intimate dimers (Lubkowski et al., 2021). The dimeric structure is necessary to build a complete active site, as residues from the C-terminal fragment of the intimate partner are necessary for substrate stabilization (Swain et al., 1993). Class 1 comprises two types of enzymes, with low (mM, type I) and high (µM, type II) substrate affinity (Srikhanta et al., 2013). Enzymes from Class 1, e.g., EcAII from *Escherihia coli*, may have metal binding sites, but they are not necessary for the catalytic activity (Borek et al., 2014; Cerofolini et al., 2019). L-Asparaginases from Class 2 belong to Ntn-hydrolases which are produced as inactive precursors and develop catalytic activity in a self-maturation cleavage process (Michalska et al., 2008). Mature Class 2 enzymes are dimers of αβ heterodimers possessing two active sites at the N termini of the β subunits. Each active site is completed by residues originating from different dimer subunits (Michalska et al., 2006; Schalk and Lavie, 2014). Class 2 L-asparaginases have a rather low substrate affinity in the range of 2–30 mM (Borek et al., 2004; Nomme et al., 2014; Ajewole et al., 2018; Janicki et al., 2023). A characteristic feature of Class 2 L-asparaginases is a sodium coordination site, formed as a “stabilization loop” in subunit α (Michalska et al., 2005; Michalska and Jaskolski, 2006; Bejger et al., 2014). This alkali metal coordination site, although separated from the substrate binding pocket (Michalska et al., 2005; Michalska et al., 2006), is crucial for the catalytic activity as it is responsible for the correct positioning of the nucleophilic Thr at the N terminus of subunit β. A subgroup of Class 2 L-asparaginases are activated by a potassium cation, which is coordinated in an “activation loop”, again away from the active site (Bejger et al., 2014). Upon K^+^ binding the enzyme is switched from off to on, with a key Arg residue assuming its productive, substrate-binding conformation.

**FIGURE 1:**
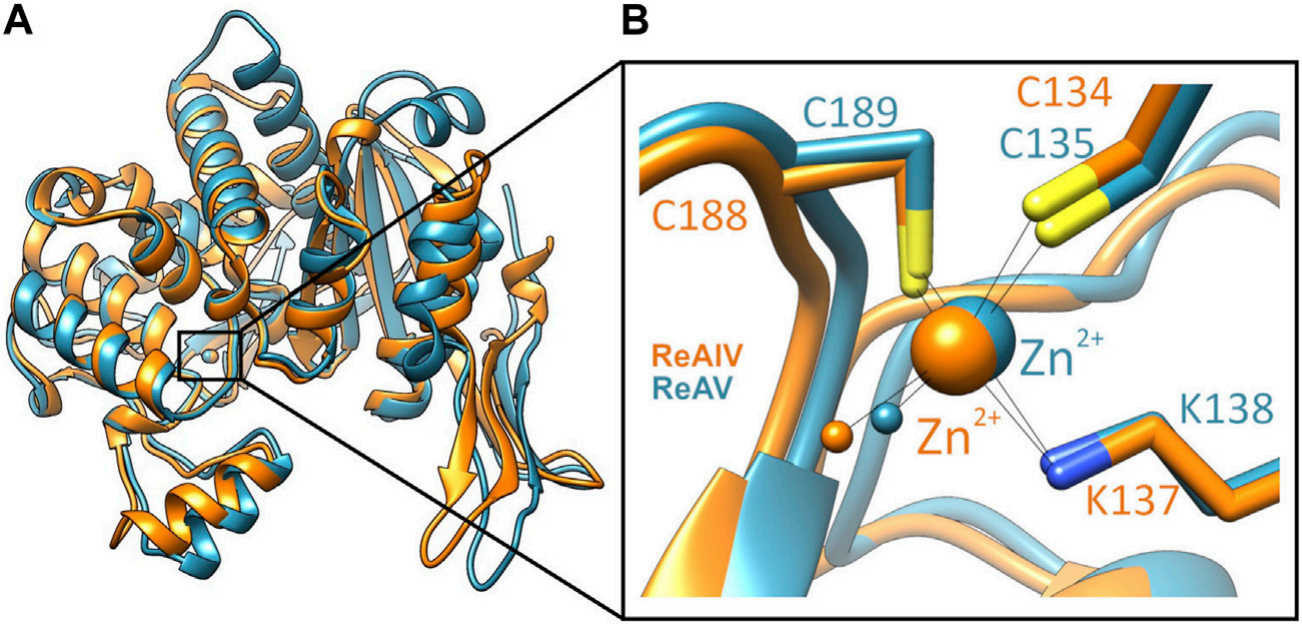
**(A)** Superposition of single subunits of ReAIV (PDB ID 8cly, orange) and ReAV (7os5, blue), with zoom-in **(B)** on the Zn^2+^ binding sites. The complete proteins are homodimeric, comprised of two such subunits.

Among the three structural Classes of asparaginases, Class 3 is the most mysterious group. It is prominently represented by two isoforms, ReAIV and ReAV, derived from *Rhizobium etli*, a nitrogen-fixing bacterial symbiont of the legume common bean. ReAIV is constitutive and thermostable, while ReAV is inducible and thermolabile, the denaturation temperatures being, respectively, 72°C and 51°C (Loch et al., 2023). The level of sequence homology between ReAIV and ReAV is rather low, with ∼31% identity and ∼45% similarity, and both proteins have no sequence similarity to other known enzymes with this activity. Despite their sequence differences, the two isoforms show high structural similarity, with the caveat that the longer ReAV sequence (367 vs. 335 residues) has additional structural elements (such as longer loops) that make it a less compact protein (Loch et al., 2023) (Figure 1A). The crystal structures of both enzymes revealed that their unusual active site contains a highly specific Zn^2+^ binding site, formed by a Lys and two Cys residues (Figure 1B), adjacent to two conspicuous Ser-Lys tandems (Loch et al., 2021; Loch et al., 2023). The ReAV and ReAIV enzymes are dimeric but, unlike in Class 1 and 2, the catalytic center is structurally composed of residues from one subunit only. A PISA analysis (Krissinel and Henrick, 2007) also suggests that the free energy of subunit interaction in ReAV and ReAIV, although still significant, is lower than in the case of the other two Classes (Table 1).

**TABLE 1:** Comparison of free energy (ΔG) of interaction between protomers of PDB representatives (PDB ID in parentheses) of 3 structural Classes of L-asparaginases, according to PISA PDB (Krissinel and Henrick, 2007).

Class	1	2	3
ΔG [kcal/mol] (protein; PDB ID)	−18.5 (EcAII; 6V23)	−40.5 (EcAIII; 2ZAL)	−10.3 (ReAIV; 8OSW)
			−12.5 (ReAV; 7OS5)

The impact of zinc on *Rhizobium* asparaginases was first studied by Moreno et al. (2012), where the inhibitory effect of 1 mM Zn^2+^ in phosphate buffer was tested. Those authors overlooked, however, that at such concentration zinc precipitates as insoluble phosphate. In the following crystallographic work on ReAV (Loch et al., 2021), we provided the first structural evidence that ReAV is a zinc metalloprotein. The use in the activity assays of high micromolar zinc concentration (in Tris-HCl buffer pH 8.0), which is about the maximum Zn^2+^ concentration achievable in a bacterial cell (100 µM), lowered the enzyme activity by 29%. That work (Loch et al., 2021), however, did not fully elucidate the role of the zinc ion in the catalytic activity. It was demonstrated, though, that treatment of the ReAV protein with a strong zinc chelator (TPEN) did not affect the enzyme kinetics. In a subsequent paper (Loch et al., 2023), using uniform buffer conditions (10 mM carbonate buffer pH 9.0 at 37°C without additional Zn^2+^) and the ITC-MIM method, we determined the kinetic parameters (K_M_, k_cat_) of ReAV and ReAIV to be 2.7 mM, 292 s^-1^ and 1.3 mM, 411 s^-1^, respectively. This indicates a rather low substrate specificity of these isoforms, but shows ReAIV to be more than two times as efficient as ReAV at those conditions. We also reported that the optimum pH of both *R. etli* L-asparaginases is 9–11 (Loch et al., 2021; Loch et al., 2023), which strongly distinguishes these enzymes from other microbial asparaginases, which have optimum pH at 7–9 (Chand et al., 2020; Muneer et al., 2020).

The aim of the present work is to fill the missing gaps in the biochemical characterization of the *R. etli* Class 3 L-asparaginases, i.e., to study the impact of factors other than pH, notably of buffering agents, salts, and temperature on their activity and kinetic parameters. In this way, we wanted to identify factors with the highest impact on enzyme activity, hoping that controlling them should allow us to arrive at reproducible kinetic results. We have updated the published kinetic parameters (Loch et al., 2023), by determining them at more optimal conditions, especially taking into account the optimal zinc concentration. We also compare the strength of zinc binding by ReAIV and ReAV, and focus on the role of zinc and other divalent metal cations in the catalysis. Finally, we are reporting a possible non-physiological substrate.

## 2 Results and discussion

### 2.1 Impact of the buffering agents

**FIGURE 2:**
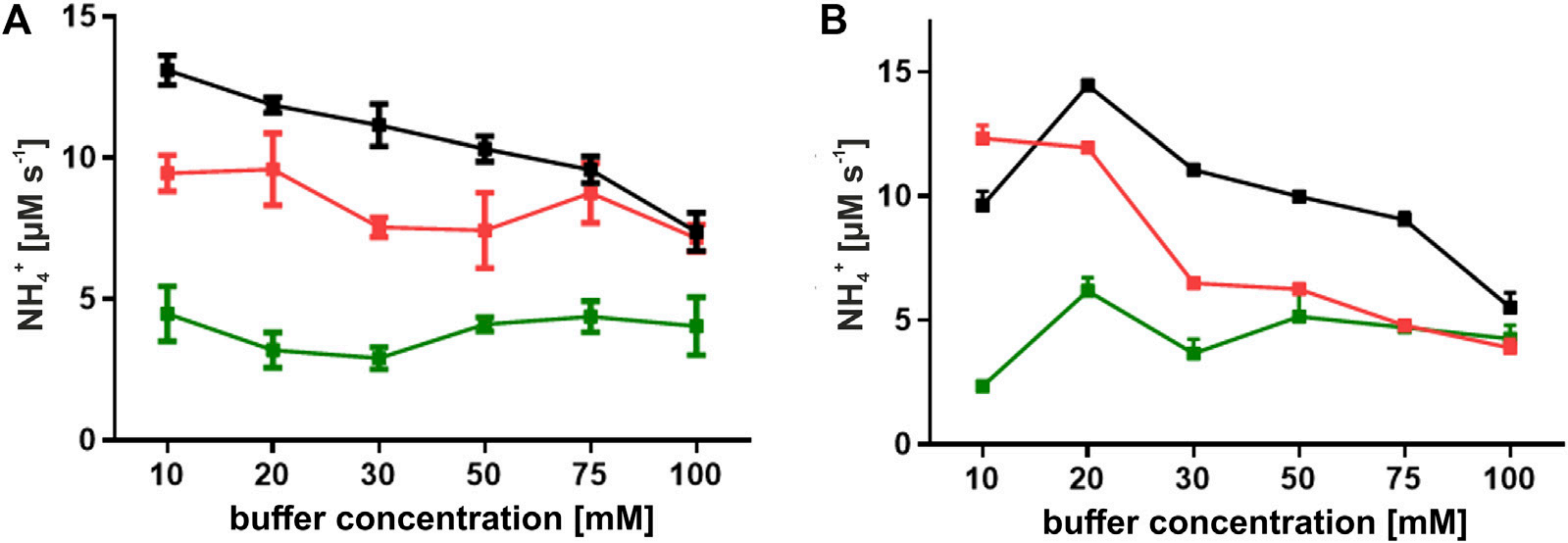
Effect of buffer concentration on the activity of ReAIV **(A)** and ReAV **(B)**. Enzyme activity was evaluated in three buffer systems: phosphate (green), carbonate (red), and Tris-HCl (black), at concentrations ranging from 10 to 100 mM in the presence of 10 mM L-asparagine. The values on the graphs represent means from three replicates with their standard deviations (±SD) shown as error bars.

The optimal pH range for both ReAIV and ReAV was determined by Loch et al. (2023) to be 9–11, with the highest activity at pH 11. In those studies, we observed a sharp increase of the enzymatic activity between pH 8 and 9, while beyond pH 9 the activity leveled out. Thus, in the present study we decided to carry out the activity and kinetic assays at pH 9.0. Doing so, we compared the activity of ReAIV and ReAV in three buffering agents, which retain their buffering capacity at pH 9.0, namely, in phosphate, carbonate and Tris-HCl buffers, at several buffer concentrations. The plots presented in Figure 2 show that both enzymes work with the highest activity in the Tris-HCl buffer, at 20 mM in the case of ReAV and 10 mM in the case of ReAIV. Both enzymes perform slightly worse in the 10 mM carbonate buffer, whereas in the phosphate buffer the activity is significantly lower at each buffer concentration. Interestingly, in the phosphate buffer there is no strong correlation between enzyme activity and buffer concentration, whereas the effect of the carbonate buffer is different, with a significant inhibitory effect at higher buffer concentrations, and the highest enzyme activity recorded at 10 mM carbonate. We also found out that increasing the carbonate concentration weakens the substrate specificity (i.e., elevates the K_M_; data not shown). A similar observation was made for Tris-HCl. However, in this case the inhibitory effect is most likely played by the chloride anion (see section 2.4).

**FIGURE 3:**
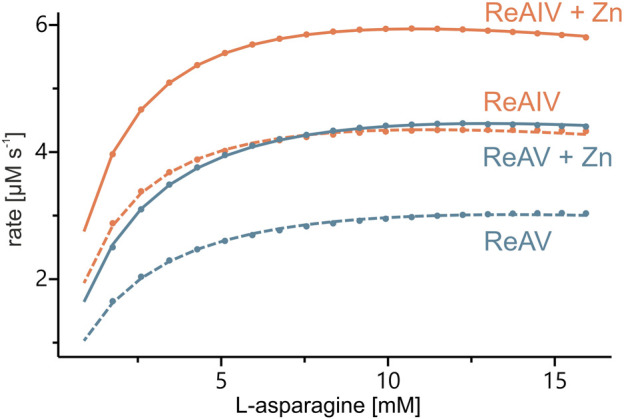
Sample kinetic curves obtained by ITC-MIM experiments in Tris-HCl buffer pH 9.0, without (dash lines) and with (solid lines) zinc supplement (1 or 2.5 μM, respectively) to 10 nM of ReAIV (orange) or ReAV (blue).

Tris-HCl and the carbonate buffer were selected for the determination of the enzyme kinetics parameters collected in Table 2 and Figure 3. One can observe that the buffering agent does not affect the K_M_ values (when measured without Zn^2+^ addition) to any significant degree; however, the turnover number k_cat_ is by ∼100 s^-1^ higher in Tris-HCl for both isoforms. It would seem that Tris-HCl pH 9.0 is the best choice for the biochemical studies of the ReAIV and ReAV enzymes, but unfortunately it is not a universally superior buffer, because: i) it slightly reduces the substrate specificity in the presence of zinc ions when compared with the carbonate buffer, probably due to the metal chelating properties of Tris (Table 2); ii) it exhibits strong complex formation properties with some of the studied transition metals, such as copper (II); iii) it has limited compatibility with Nessler reagent assays, as precipitation at higher substrate concentration is observed; iv) as its pH is temperature-dependent and the activity of the studied enzymes is strongly pH-dependent, this buffer is unsuitable for temperature-dependent studies. For the above reasons, some of our biochemical studies were carried out in a carbonate environment. Nevertheless, we present the kinetic parameters in both buffering conditions (Tris-HCl and carbonate) to establish the highest achievable enzyme efficiencies of ReAIV and ReAV. Additionally, as we discovered that TCEP inhibits the activity of both isoforms (Supplementary Figure S1), we also excluded this additive from the reaction buffers.

**TABLE 2:** Kinetic parameters for ReAIV and ReAV isoforms from ITC-MIM experiments in different buffering systems at 37°C. In the bottom part of the table (shaded), the kinetic parameters in the presence of low zinc concentration, 1.0 µM (ReAIV) or 2.5 µM (ReAV), are shown. The presented values are averages from three separated experiments with their ±SD errors.

ReAIV				ReAV		
Buffer	K_M_ [mM]	k_cat_ [s^-1^]	k_cat_/K_M_ [s^-1^mM^-1^]	K_M_ [mM]	k_cat_ [s^-1^]	k_cat_/K_M_ [s^-1^mM^-1^]
Carbonate	1.7 ± 0.0	428 ± 13	246 ± 8	2.6 ± 0.2	327 ± 14	126 ± 14
+ Zn^2+^	1.3 ± 0.1	543 ± 17	407 ± 43	1.9 ± 0.1	392 ± 34	203 ± 3
Tris-HCl	1.6 ± 0.1	546 ± 21	348 ± 5	2.7 ± 0.1	416 ± 3	154 ± 7
+ Zn^2+^	1.5 ± 0.1	770 ± 17	526 ± 34	2.1 ± 0.2	603 ± 24	294 ± 31

### 2.2 ReAIV is stronger Zn^2+^ binder than ReAV

**FIGURE 4:**
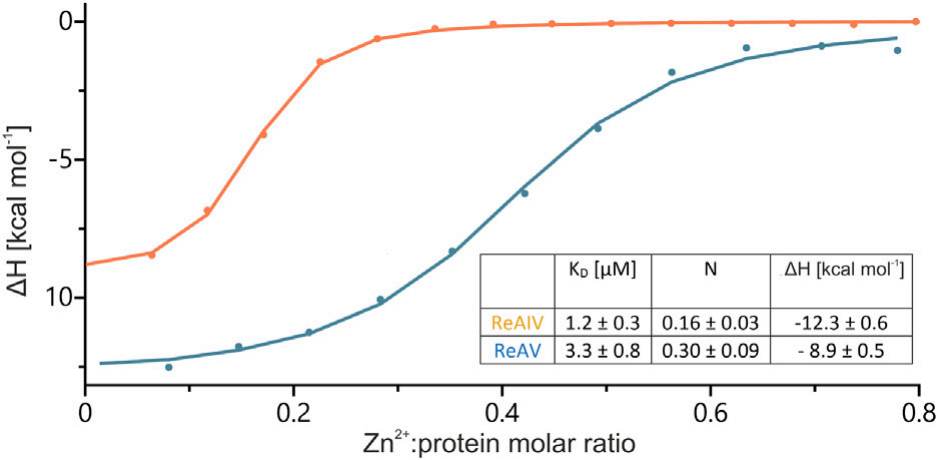
Integration of heat peaks from representative ITC data, with the best fit of ‘one set of sites’ model, obtained after titrations of ∼168 µM ReAIV (orange) or ∼149 µM ReAV (blue) with 1 or 0.9 mM Zn^2+^, respectively. In the inset, the Zn^2+^ binding parameters obtained in two separate experiments are collected together with their ± SD errors.

The structural studies of ReAV and ReAIV have clearly indicated that the zinc center is almost identical in both isoforms (Loch et al., 2021; Loch et al., 2023), regardless of their low sequence identity. In Loch et al. (2021) we determined the K_D_ of Zn^2+^ binding by the ReAV protein as 2.7 ± 0.9 µM. In that previous work we had used different reagents and different bacterial competent cells for target protein expression. In order to reliably compare the strength of zinc binding by the ReAIV and ReAV isoforms, in the present work we first attempted to recreate the TPEN zinc removal procedure and Zn^2+^ microcalorimetric titration for the ReAV protein. The re-determined K_D_ value is 3.3 ± 0.8 µM (Figure 4), which agrees within the margin of error with the previous value (Loch et al., 2021). By the same recreated procedure we then found that ReAIV binds Zn^2+^ ions nearly three times more strongly, with a K_D_ of 1.2 ± 0.3 µM. Interestingly, in both cases the experimentally determined stoichiometry of the zinc complexes *N*) is fractional (but still two times higher for ReAV compared to ReAIV), whereas in Loch et al. (2021) the reported *N* value for ReAV was close to 1. The reason for this disagreement could be either only fractional contribution of bioactive protein due to misfolding, or the possibility that we were not able to remove the zinc ion from the protein completely, despite reproducing the earlier procedure and even applying a higher molar excess of TPEN than in the previous work. The higher *N* value for ReAV could suggest that it was easier to remove zinc during the preparation of the apo form of ReAV, which is logical considering the fact that ReAV is a weaker Zn^2+^ binder than ReAIV. Nevertheless, in view of the problems with obtaining an integer (i.e., *N* = 1) and reproducible stoichiometry when titrating the enzymes with Zn^2+^ ions after the TPEN procedure, we must question the applicability of the “TPEN approach” to studying the zinc role in enzymatic activity. On the other hand, we cannot rule out that in control kinetic experiments, in which the enzyme was not pretreated with TPEN, the protein was not fully saturated with zinc, since as the protein is diluted to nanomolar concentrations, the equilibrium is shifted towards the dissociated components. Therefore, to test the role of zinc in the present study, we investigated the effects of: i) low physiological and optimal zinc concentration and ii) weakening of zinc binding by site-directed point mutations (e.g., of the lysine involved in zinc coordination), on the kinetic parameters of the enzymes.

### 2.3 Optimal Zn^2+^concentration boosts the turnover number and substrate specificity

**FIGURE 5:**
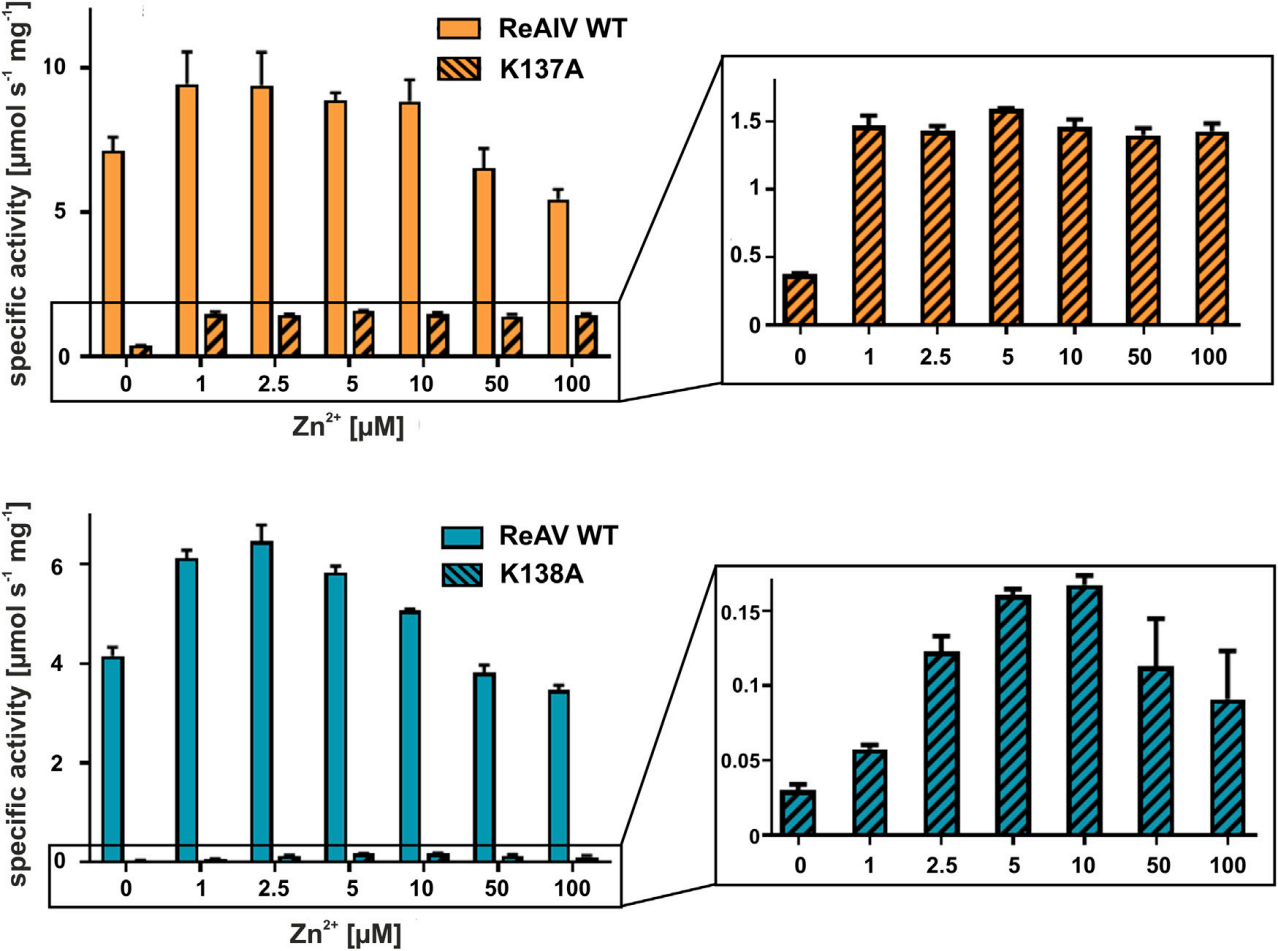
Effect of ZnCl_2_ on ReAIV WT (orange, plain) and its K137A mutant (orange, stripes), and ReAV WT (blue, plain) and its K138A mutant (blue, stripes) activity determined in 20 mM Tris-HCl buffer pH 9.0 by the Nessler reaction. Specific activity is marked on the *Y*-axis to show the scale of the differences between the maximum activities achieved by the isoforms and their mutants. The zinc profile of the mutants was zoomed in insets for better visualization of the optimum Zn^2+^ concentration. The graphs show mean values obtained from three determinations, with their ± SD as error bars.

With the above misgivings in mind, we decided to first optimize the zinc concentration (Figure 5) and found that low micromolar Zn^2+^ level improves the enzymatic activity relative to a “non-supplemented'' preparation by us much as 32% in the case of ReAIV (for 1 µM Zn^2+^) and as much as 56% in the case of ReAV (for 2.5 µM Zn^2+^). These experiments also showed that under the tested conditions ReAIV was more sensitive to the highest 100 µM Zn^2+^ concentration, which inhibited the activity by 24% compared to the initial value, than ReAV, which showed a threefold lower (7%) inhibition. The lower sensitivity to high zinc concentrations of ReAV may be a consequence of its weaker zinc binding, compared to ReAIV. This experiment clearly showed a significant increase of enzymatic activity in the presence of low zinc level in the reaction buffer. Next, we checked the influence of the optimal zinc content on the kinetic parameters of the isoforms (Table 2; Figure 3) and found that it significantly improves both the substrate specificity and turnover numbers of both enzymes. For instance, in the Tris-HCl buffer, Zn^2+^ at its optimal concentration improves the enzymatic efficiency of ReAV by 91% and of ReAIV by 51% (Figure 3; Table 2).

It is worth noting that the optimum Zn^2+^ concentration for both isoforms is close to the K_D_ values of zinc binding. A K_D_ of 1.2 or 3.3 µM indicates a rather high affinity, albeit not the highest achievable in biological systems. This means that the Zn^2+^ ion exists in a dynamic equilibrium with its protein complex. Supplying the buffer with this cation at the physiological level, shifts the equilibrium toward the metal-bound state of the protein. Consequently, in all subsequent assays of Class 3 L-asparaginase activity, we consistently maintained the optimal zinc concentration in the buffer solutions.

### 2.4 The impact of Zn^2+^ on enzyme kinetics is highlighted by mutants with impaired Zn^2+^ coordination

In both *R. etli* L-asparaginase isoforms, the zinc coordination site is formed by two cysteine residues, one water molecule and a lysine residue at position 137/138 in ReAIV/ReAV numbering (Figure 1B), (Loch et al., 2021; Loch et al., 2023). In order to evaluate and compare the effect of perturbed zinc coordination on the kinetics of both isoforms, we studied their site-directed mutants with the lysine changed to alanine. First, we searched for the optimum zinc concentration for each mutant (Figure 5). Next, we determined the kinetic parameters without and with optimum Zn^2+^ concentration (Table 3). It should be noted that the same mutation, even in the presence of optimal zinc concentration, causes as much as almost 40-fold decrease in ReAV activity, while less than 7-fold decrease in the case of ReAIV, indicating that the amino group of the “missing” Lys residue is more “important” in the case of ReAV. One can also conclude that zinc supply itself does not restore the “wild type activity”. Nevertheless, the K→A mutants of both isoforms appeared to be much more responsive to Zn^2+^ in solution than the wild type proteins, as zinc increased their activity (in comparison to non-zinc-supplemented proteins) as much as four times in both cases (Figure 5). Of note is also the absence of any inhibitory effect of high zinc concentrations, which was observed for the wild-type forms, as well as a shift of the optimum effect to higher zinc concentrations, which may be a consequence of the weaker zinc binding by these mutants. Zinc has even a more pronounced effect on the kinetic parameters of these mutants than on the wild-type enzymes. In the case of the ReAIV K137A mutant, addition of Zn^2+^ causes an almost 10-fold increase of enzymatic efficiency (increased substrate specificity from K_M_ of 4.7 to 2.3 mM, with simultaneous 5-fold increase of the turnover number from 35 to 158 s^-1^). On the other hand, addition of optimal zinc amount to the corresponding ReAV mutant causes a 7-fold efficiency increase, but this time mainly due to a significant increase in substrate specificity (2.2–0.5 mM). The increased sensitivity of the mutants with impaired Zn^2+^ binding to the presence of this ion in the solution emphasizes the role of Zn^2+^ in substrate recognition.

**TABLE 3:** The effect of K→A mutation on the kinetic parameters of ReAIV and ReAV and the effect of optimal Zn^2+^ concentration on these parameters, determined in 10 mM Tris-HCl buffer pH 9.0 by ITC-MIM. The values are averages of two (in the case of mutants) or three (WT enzymes) measurements, with their ±SD errors.

	ReAIV				ReAV			
	WT	+ Zn^2+^	K137A	+ Zn^2+^	WT	+ Zn^2+^	K138A	+ Zn^2+^
K_M_ [mM]	1.6 ± 0.1	1.5 ± 0.1	4.74 ± 0.04	2.3 ± 0.1	2.7 ± 0.1	2.1 ± 0.2	2.1 ± 0.1	0.5 ± 0.1
k_cat_ [s^-1^]	546 ± 21	770 ± 17	35 ± 4	158 ± 6	416 ± 3	603 ± 24	4.8 ± 0.4	6.5 ± 0.1
k_cat_/K_M_ [s^-1^mM^-1^]	348 ± 5	526 ± 34	7 ± 1	70 ± 1	154 ± 7	294 ± 31	2.2 ± 0.1	14 ± 3

### 2.5 The impact of divalent metal cations: varied resistance of ReAIV and ReAV to Hg^2+^, Cu^2+^, Cd^2+^, and Ni^2+^ ions

**FIGURE 6:**
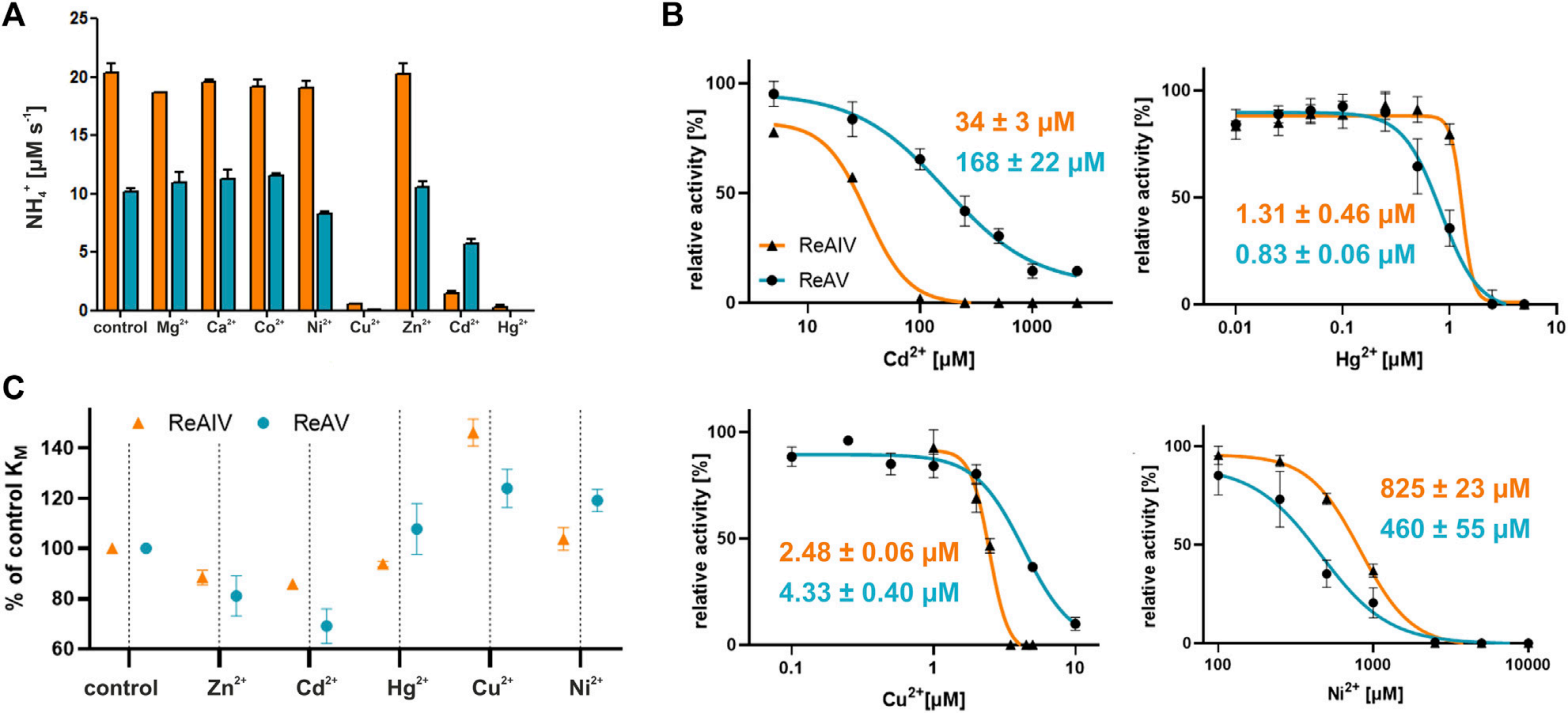
**(A)** The effect of divalent metal cations on the activity of ReAIV (orange) and ReAV (blue) after 5 min preincubation with chloride salt of each metal at the concentration of 100 μM, as determined by the Nessler method in 10 mM Hepes buffer pH 8.5. The graphs represent averages of two determinations, with their ± SD standard deviations shown as error bars. **(B)** The IC50 parameters determined for preselected cations with inhibitory potential for ReAIV (orange) and ReAV (blue), the error bars indicate ±SD of the mean from three replicates **(C)** The effect of 2.5 μM (or 0.2 μM in the case of Hg^2+^) addition of preselected cations on the K_M_ parameter of ReAIV and ReAV expressed as % relative to the K_M_ value without metal addition, the error bars indicate ±SD of the mean from two replicates.

We also conducted a survey of the effects of divalent metal cations on ReAV activity, assuming that other transition metals, such as Hg^2+^, Cd^2+^, Cu^2+^, Co^2+^, or Ni^2+^ could compete with zinc for the binding site. We also tested the effect of Mg^2+^ and Ca^2+^. The Nessler activity assay was performed for each metal at 100 µM concentration (Figure 6A). It should be stressed that we took into account the low solubility of carbonates of some transition metals, as well as the strong complexation of the Cu^2+^ ion in Tris-HCl buffers (Nagaj et al., 2013), leading in our case to masking of the inhibitory properties of copper. To circumvent this difficulty, these assays were ultimately performed in a Hepes pH 8.5 buffer. The screens show that both isoforms have no activity in the presence of 100 µM mercury or copper ions. Additionally, ReAV exhibits ∼20% loss of activity in the presence of nickel ions and ∼40% loss of activity in the presence of cadmium ions. On the other hand, the activity of ReAIV is strongly diminished (by more than 90%) by cadmium ions, but is not affected by nickel. For a better comparison of the differences in response to Hg^2+^, Cd^2+^, Cu^2+^, Co^2+^, and Ni^2+^, we additionally determined the IC50 values for both isoforms (Figure 6B). The most significant difference between the two enzymes is in the response to Cd^2+^, as ReAIV is five times more sensitive to this metal ion than ReAV (IC50 34 vs. 168 µM). Although Hg^2+^ has the highest inhibitory effect on both isoforms among the tested metal ions, ReAIV is more resistant to this ion (IC50 ∼1.3 µM vs. 0.8 µM). Cu^2+^ is also a strong inhibitor of rhizobial asparaginases, although ReAV is less sensitive than ReAIV (IC50 ∼4.3 µM vs. 2.5 µM). The nickel ion has the lowest inhibitory effect on both isoforms, although it inhibits ReAV more strongly (IC50 ∼460 µM) than ReAIV (IC50 ∼825 µM).

We also studied the effect of the selected metal ions on substrate specificity (Figure 6C). We observed that Cd^2+^ improves the substrate specificity of ReAV to a greater extent (and even more than Zn^2+^) than in the case of ReAIV, when compared to a protein sample without metal addition. Hg^2+^ improves slightly the specificity of ReAIV, but not of ReAV. Cu^2+^ reduces the substrate specificity of both isoforms, but does it more extensively in the case of ReAIV. On the other hand, Ni^2+^ has negligible impact on the K_M_ value of ReAIV but reduces the substrate specificity of ReAV.

Additionally we scanned the thermal stability of both isoforms in the presence of equimolar concentrations of the above cations in a 20 mM Hepes buffer (Table 4). We found that the melting temperatures of untreated ReAIV and ReAV in such conditions are 49.4°C and 70.0°C respectively, which are slightly below the previously determined T_m_ values (51°C and 72°C), which, however, had been determined in 20 mM Tris-HCl. As it has been already shown by the crystal structures, multiple chloride ions can be bound to rhizobial asparaginases, and these ionic and H-bonding interactions could lead to higher thermal stability in Tris-HCl buffer. We observed a slight improvement of these melting temperatures in the presence of every metal ion tested, except Cu^2+^ in the case of ReAIV and Hg^2+^ in the case of ReAV, which lowered the T_m_ values by 2.1°C and 0.4°C, respectively. Interestingly Cu^2+^ has no such adverse effect on ReAV stability and improves its T_m_ by 1.6°C, whereas Hg^2+^ has a similar positive effect on ReAIV stability, increasing its T_m_ by 1.4°C. These observations are consistent with the IC50 differences between ReAIV and ReAV determined for Hg^2+^ and Cu^2+^, as well as with the effect of these cations on the substrate specificities of both isoforms. We also discovered that cadmium stabilizes the ReAV protein to even greater extent than Zn^2+^ (T_m_ improvement by 2.5°C and 1.5°C, respectively), whereas in the case of ReAIV these cations increase the T_m_ by the same amount (1.9°C and 2.0°C, respectively). These findings correlate with the fact that Cd^2+^ improves the substrate specificity of ReAV to a greater extent than Zn^2+^, a phenomenon not observed for ReAIV, and with IC50 of Cd^2+^ five times higher for ReAV than for ReAIV. Ni^2+^ has the same impact on T_m_ of ReAIV as Cd^2+^. We could not collect similar data for ReAV and Ni^2+^ because of protein precipitation.

**TABLE 4:** Melting temperatures [°C] obtained by nanoDSF measurements of ReAIV and ReAV isoforms without and in presence of equimolar concentrations of Zn^2+^, Cd^2+^, Hg^2+^, Cu^2+^, or Ni^2+^ ions in 20 mM Hepes buffer pH 8.0. Additionally, in the columns on the right, the changes in Tm after the addition of a metal ion in relation to the control sample were calculated. The values are averages from two experiments, with their ±SD errors.

	ReAIV	ReAV	ReAIV	ReAV
	Tm [°C]		ΔTm [°C]	
control	70.0 ± 0.3	49.4 ± 0.2	0	0
Zn^2+^	72.0 ± 0.1	50.9 ± 0.1	2 ± 0.3	1.5 ± 0.2
Cd^2+^	71.9 ± 0.1	51.9 ± 0.2	1.9 ± 0.3	2.5 ± 0.3
Hg^2+^	71.4 ± 0.1	49.0 ± 0.4	1.4 ± 0.3	−0.4 ± 0.4
Cu^2+^	67.9 ± 0.2	51.2 ± 0.1	−2.1 ± 0.4	1.8 ± 0.2
Ni^2+^	71.9 ± 0.1	*-*	1.9 ± 0.3	-

The selected transition metal cations differ from the physiological Zn^2+^ not only in ionic radii (e.g., the bigger Cd^2+^) but also in their preferred coordination geometry in case of (e.g., Ni^2+^, Hg^2+^ and Cu^2+^) (Rulisek and Vondrasek, 1998). Since Zn^2+^ most probably takes part in substrate recognition, as its presence improves substrate specificity (a phenomenon particularly pronounced in K→A mutants), replacing it with other transition metal ions may have far-reaching consequences. For instance, an exchange of the metal co-factor to Cd^2+^ might result in stronger substrate binding and in improved substrate specificity (as in the case of ReAV with Cd^2+^). On the other hand, the metal exchange could also retard product release, resulting in lower k_cat_ and slower enzyme activity. The altered coordination geometry (compared to Zn^2+^), especially in the case of Cu^2+^, degraded the substrate specificity of both isoforms, probably due to incorrect orientation of the substrate molecule. Further structural studies of both isoforms in complex with selected transition metal cations should illuminate their inhibition mechanism.

The different response of ReAIV and ReAV to several metal ions is puzzling and the question arises about the role of the inducible isoform ReAV with such distinct features. Rhizobia are known to help the plant symbiont survive abiotic stress caused by heavy metal contamination through their extensive heavy metal tolerance/resistance mechanism based on active efflux and sequestration, which also plays an important role in phytoremediation (Jach et al., 2022). During such stress, related, for example, to soil acidification (which increases the availability of free heavy metal ions), a protein isoform with different metal binding properties may be crucial for the survival.

### 2.6 The oligomeric state of the proteins

**FIGURE 7:**
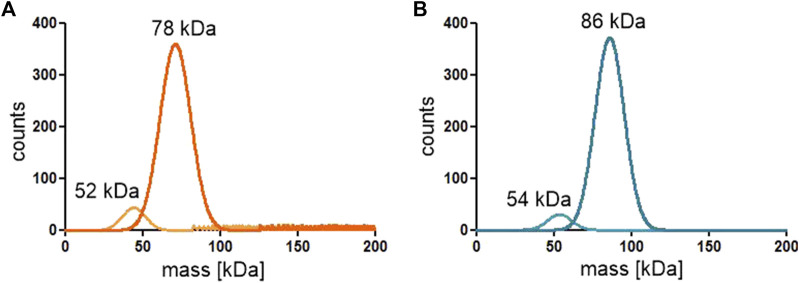
Distribution of particle size of the ReAIV **(A)** and ReAV **(B)** proteins measured at 25–50 nM concentration by mass photometry.

Since the active site of both ReAV and ReAIV is near the dimer interface (Loch et al., 2021; Loch et al., 2023), we decided to investigate whether the dimeric state is stable, and whether the kinetic assay conditions (low nanomolar protein concentration, diluted carbonate buffer, absence or presence of Zn^2+^ and/or Mg^2+^ ions) could change it. To check this, we carried out mass photometry experiments. The measurements showed that under the conditions of experiment (with protein at ∼50 nM concentration) both proteins remain in the dimeric state with very low tendency to dissociate into monomers, as illustrated by the Gaussian distribution of particle size (Figure 7). This is consistent with the free enthalpy of dimerization (ΔG) calculated by the PISA server (Krissinel and Henrick, 2007) for the highest resolution crystal structures of both proteins (Table 1). If we then calculate the theoretical dissociation constant K_D_ for both dimers from the ΔG = RTlnK equation, we find out that at 25°C the K_D_ for ReAIV is 28 nM, whereas for ReAV 0.7 nM. This indicates that ReAV forms a stronger dimer than ReAIV.

### 2.7 Chloride ion inhibits rhizobial asparaginases to varying degrees

**FIGURE 8:**
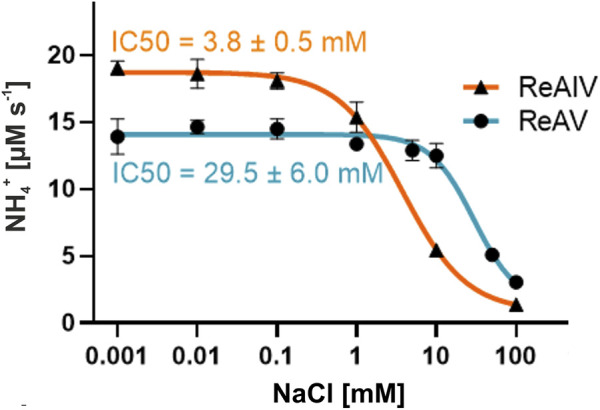
Chloride ion inhibition of ReAIV and ReAV activity demonstrated by the IC50 values. The rates of NH_4_^+^ production were determined by the Nessler method in 10 mM carbonate buffer pH 9.0, supplemented with 1 or 2.5 µM ZnCl_2_ for ReAIV or ReAV, respectively. Data pointes represent averages from four determinations, with their ± SD shown as error bars.

To explain the effect of the chloride ions, we established that the IC50 value of NaCl (Figure 8) is ∼8 times lower for ReAIV than for ReAV (3.8 vs. 29.5 mM) at the same assay conditions. We also determined the K_M_/k_cat_ kinetic parameters for both isoforms at 20 mM NaCl concentration (1.6 mM/174 s^-1^ for ReAIV and 3.9 mM/341 s^-1^ for ReAV), demonstrating that with increased NaCl concentration, the efficiencies of the two proteins become closer to each other, and that the chloride anion affects more significantly the k_cat_ parameter of ReAIV and the K_M_ parameter of ReAV. This feature of the inducible isoform ReAV could be of special relevance during salinity and drought abiotic stresses. Negatively charged anions (such as Cl^−^) most likely block the active site by docking in the site reserved for the negatively-charged α-carboxylate group of the substrate molecule. Such an effect is well known from other asparaginases, e.g., Class 2, where an Cl^−^ is also known to bind instead of the substrate (Michalska et al., 2008).

**FIGURE 9:**
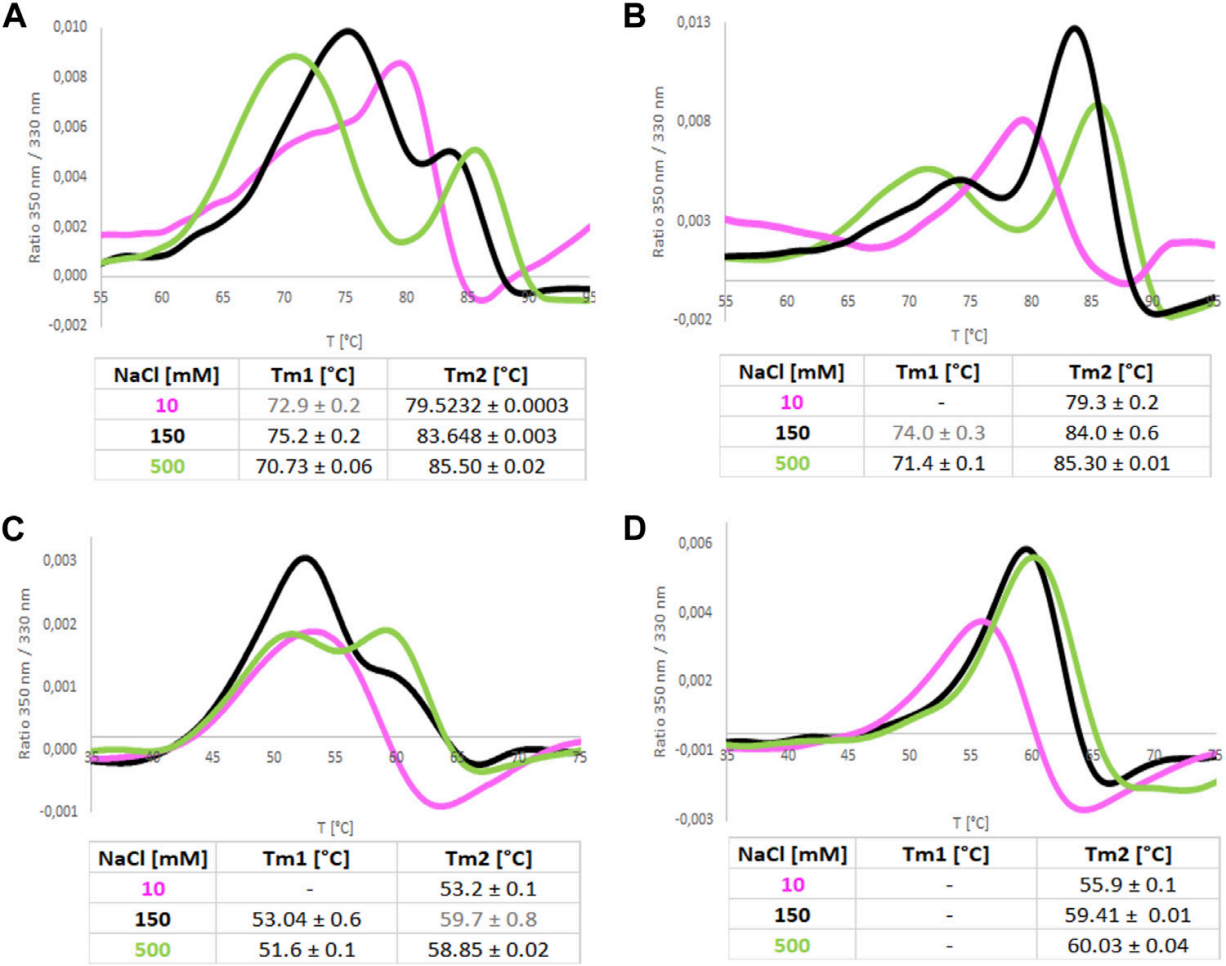
Melting curves recorded by NanoDSF for ReAIV **(A,B)** and ReAV **(C,D)** proteins in 50 mM Tris-HCl buffer pH 8.0 at 10, 150, and 500 mM NaCl, with **(B,D)** and without **(A,C)** equimolar concentration of Zn^2+^. The tables below collect the mean values from 4 replicates with their ± SD errors.

In addition, we recorded nanoDSF melting scans of both isoforms in increasing NaCl concentration (Figure 9). They showed that increased NaCl concentration promotes a two-step melting trajectory. This effect is stronger in the case of ReAIV, where T_m_1 appears already at 10 mM NaCl. Hypothetically, the first peak could represent the dissociation of the dimer and the subsequent second peak the denaturation of the monomer. As already discussed in section 2.6, ReAIV forms a weaker dimer than ReAV, therefore early dimer dissociation of that isoform might occur already at low NaCl concentration, while for ReAV it is observed only at 150 and 500 mM NaCl. Interestingly, the presence of Zn^2+^ cations completely changes the course of the melting scan of ReAV from a two-step to one-step at 150 and 500 mM NaCl concentration. In the case of ReAIV, Zn^2+^ changes the melting scan to one-step only in the presence of a low concentration of NaCl (10 mM). Thus, we postulate that the Zn^2+^ ions could protect the protein from monomerization at high temperature and at high NaCl concentration. Moreover, one can observe the increase of T_m_2 with the increasing NaCl concentration. The highest achievable T_m_2 values are more than 85°C for ReAIV and 60°C for ReAV in 500 mM NaCl (in both cases more than 10°C than measured in a salt-free buffer), which proves that thermal stability of a structure is not equivalent to better activity in case of high NaCl concentration.

### 2.8 The influence of temperature on enzyme efficiency

**FIGURE 10:**
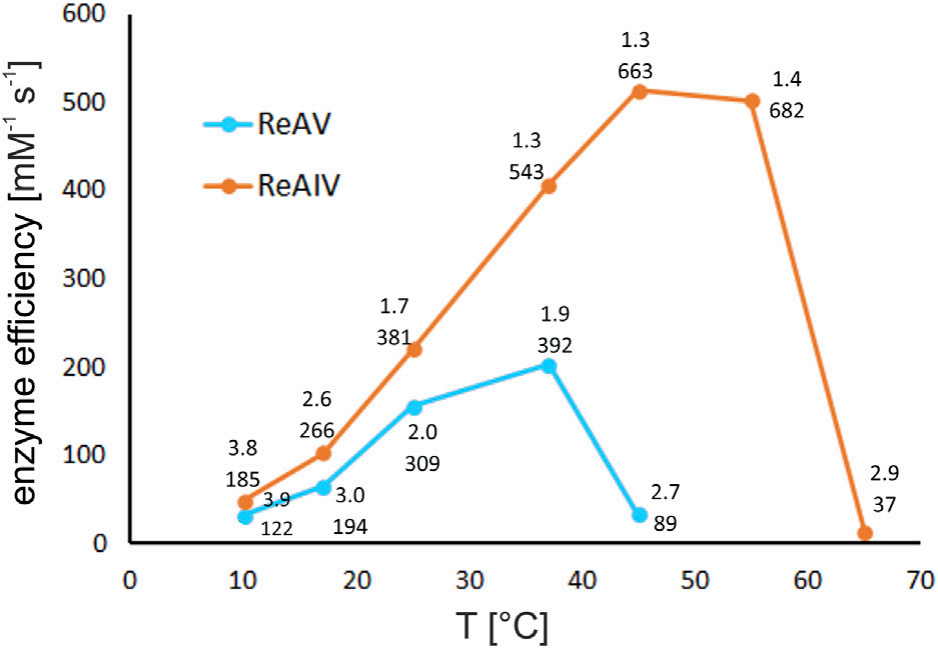
The effect of temperature on kinetic efficiency of ReAIV and ReAV, determined by single ITC-MIM experiments in 10 mM carbonate buffer pH 9.0 at 10, 17, 25, 37, 45, 55, and 65°C, in the presence of optimal zinc concentration. The kinetic parameters K_M_ [mM] (upper) and k_cat_ [s^-1^] (lower), are placed near each temperature point.

Utilizing the thermostating capability of the microcalorimeter, we studied the enzymatic rate of both isoforms at several temperatures, namely, 10, 17, 25, 37, 45°C, and additionally at 55°C and 65°C for ReAIV (Figure 10). It is noteworthy that at lower temperatures (10°C and 17°C) both enzymes operate with similar efficiency, and ReAIV is only slightly more efficient than ReAV. At elevated temperatures (25°C and above), the thermostable ReAIV enzyme is significantly more efficient than the thermolabile ReAV, which reaches its optimum at 37°C (k_cat_ = 392 s^-1^, K_M_ = 1.9 mM). The enzyme efficiency of ReAV drops drastically at 45°C and of ReAIV at 65°C, which in both cases is about ∼7°C before their respective melting points of 51°C and 72°C.

Interestingly, the temperature of 45°C, at which the efficiency of ReAV drops dramatically, is also the optimal temperature for ReAIV activity, where the kinetic parameters of this enzyme (in carbonate buffer and with Zn^2+^ supplementation) are as good as k_cat_ = 663 s^-1^ and K_M_ = 1.3 mM (efficiency of 514 mM^-1^ s^-1^). The thermal profile of ReAIV is quite broad, as further increase of temperature to 55°C does not affect its efficiency very much, and even causes further increase of the turnover number with simultaneous slight drop of substrate specificity (k_cat_/K_M_ of 682 s^-1^/1.4 mM).

Regardless of the different optimal operating temperature of the two enzymes (37°C vs. 45°C for ReAV vs. ReAIV, respectively), most measurements of the kinetic parameters in this work were carried out at 37°C, with possible medicinal applications in mind. The higher melting temperature of ReAIV and the higher temperature optimum of its activity may be the consequence of the more compact structure of this isoform (Figure 1A).

### 2.9 Activity towards other substrates

Using ITC calorimetry, we found out that neither ReAIV nor ReAV produces any heat effect upon titration with 100 mM L-Gln, even at an elevated protein concentration of 1 μM, i.e., 70 times higher than in the L-asparaginase assays (Supplementary Figures S2A, B). Lack of L-glutaminase activity is a desirable feature in an L-asparaginase enzyme considered in the context of its possible use in acute lymphoblastic leukemia (ALL) therapy, as it reduces undesirable side effects of the drug. We also tested other potential substrates, such as acrylamide and urea (Saeed et al., 2018), finding that after acrylamide injection into the reaction cell containing ReAIV, but not ReAV, an observable enthalpy production can be detected (Supplementary Figures S2A, C). This exothermic effect may result from the hydrolysis of acrylamide into acrylic acid and ammonium ions, although their level is not detectable by the Nessler reaction. Even though a similar exothermic effect is not observed for BSA or ReAV (Supplementary Figure S2C), we cannot rule out that the enthalpy observed with ReAIV is the result of another phenomenon, such as the formation of an acrylamide protein adduct (Bonaventura et al., 1994). This would be consistent with the observed strong product inhibition or enzyme inactivation at substrate concentration of ∼6 mM (Supplementary Figures S2D, E). The kinetic parameters of the observed reaction, obtained from the fitting of the Michaelis-Menten equation to the data obtained before product inhibition/enzyme inactivation, are ∼5.5 mM and ∼12.6 s^-1^ (Supplementary Figure S2F). The nature of this reaction will be the subject of further research. There was no detectable heat effect with urea for both isoforms.

### 2.10 General observations regarding protein sample preparation and storage

We were initially confronted with different K_M_ values from ReAV preparation to preparation, and with the fact that diluting ReAV after SEC to 10 mM carbonate buffer resulted in a time-dependent improvement of substrate specificity and enzyme efficiency (with the highest activity observed after overnight dialysis). One of the reasons for this behavior might be a slow dissociation of some inhibitory factors derived from the bacterial culture (e.g., endotoxins). Since the expression levels of ReAV in *E. coli* cells are very high, up to 100 mg L^-1^, we wanted to investigate whether the IPTG concentration used for the induction (tested at 100, 200, 300, and 400 µM IPTG) could affect the levels of protein expression, and whether endotoxins content in the protein preparations could affect the substrate specificity of RaAV. We did not observe any correlation of IPTG concentration with endotoxin levels and with the levels of protein expression, however, we did observe some correlation between the level of endotoxins and substrate specificity (Supplementary Figure S3). We, therefore, hypothesize that the improvement of K_M_ of dialyzed samples may be, at least partly, due to the removal of endotoxins from protein preparations, as endotoxins might interfere with substrate binding. In contrast, the expression level of ReAIV is much lower, ∼10 mg L^-1^. We also established that endotoxin level in ReAIV samples is over 10 times lower than in ReAV preparations (0.15 vs. 1.7 EU/mL). We also did not observe any beneficial effect of post-purification dialysis on the kinetic parameters of ReAIV.

After numerous activity tests, we were able to establish, for both isoforms, the optimal sample storage conditions that had minimal detrimental effect on enzymatic activity. Obviously, homogeneity of the preparation is of key importance, and this is ensured by carrying SEC purification for fresh or frozen samples just before the enzymatic assays. Subsequent extensive dialysis against carbonate buffer (in case of ReAV) without NaCl addition significantly improved the kinetic parameters, possibly by ridding the sample of the inhibitory bacterial metabolites. We also noted that the ReAV protein retains its high activity for the longest time when it is kept on ice, in the presence of salt, in the SEC buffer. Dialysis against carbonate buffer should be carried out for small aliquots, just prior to the intended assay. We conclude from these observations that although the Cl^−^ anions inhibit the L-asparaginase reaction of ReAIV and ReAV (probably by docking to the substrate binding site, as discussed in chapter 2.7), they also extend the longevity of the protein, probably by stabilizing the structure via ionic interactions, as revealed by the crystal structures (Loch et al., 2021; Loch et al., 2023). High ionic strength and buffering capacity will also have a beneficial effect on protein storage, albeit not necessarily on the enzymatic performance.

## 3 Conclusion

Class 3 L-asparaginases are not only sequentially and structurally different from other known enzymes with this activity, but they also have very different optimum conditions for their performance. The ReAIV and ReAV representatives, despite having a similar fold and the same active center, have low sequence identity and even polypeptide length. The longer ReAV sequence makes the structure less compact, which has further functional consequences. Both isoforms have an optimum activity in the alkaline pH range. However, our biochemical characterization showed that the two enzymes complement each other in other aspects. At lower temperatures, they have similar efficiencies, but the temperature optimum of ReAV lies more than 10°C lower than that of ReAIV. At its temperature optimum (37°C), ReAV is two times less efficient than ReAIV. The activity of both isoforms is boosted by low and optimal zinc concentration, with ReAV activity being more responsive to zinc presence in the buffer. The extra zinc in the buffer has a significant impact on both, substrate specificity and turnover number. The improvement of K_M_ indicates a role of the metal ion in substrate recognition. ReAV binds Zn^2+^ thrice more weakly than ReAIV, but is correspondingly less susceptible to its inhibition at high concentrations. The role of the zinc ion is additionally emphasized by the low activity and efficiency of ReAIV and ReAV mutants with a crippled zinc coordination site. The two isoforms also show a different response to other transition metals: ReAIV appears to be more sensitive to Cd^2+^ and Cu^2+^ than ReAV, while ReAV is more sensitive to the presence of Hg^2+^ and Ni^2+^. ReAIV is almost eight times more sensitive than ReAV to chloride ion concentration.

The above different features of the two enzymes can be crucial for survival at abiotic stress conditions, such as drought, salinity, or heavy metal contamination. The constitutive ReAIV is undoubtedly a more efficient asparaginase than the inducible ReAV, but under suboptimal conditions, ReAV is an excellent candidate as an auxiliary enzyme, matching the efficiency of ReAIV in the elevated presence of Cd^2+^, Cu^2+^, or Cl^−^ ions. The present thorough biochemical analysis of both isoforms, when combined with physiological studies, may help answer the intriguing questions about the reason for the coexistence of a constitutive and inducible L-asparaginase in *Rhizobium etli*.

## 4 Materials and methods

### 4.1 Protein preparation

DNA constructs for expression of the constitutive thermostable (ReAIV) and inducible thermolabile (ReAV) WT (wild type) *R. etli* L-asparaginases were obtained as described previously (Loch et al., 2021; Loch et al., 2023). Production of the ReAV and ReAIV proteins was conducted in *E. coli* strain BL21 Gold (DE3). The bacterial cultures were grown at 37°C to middle logarithmic phase and expression was induced with 0.3 mM IPTG, while keeping the bacterial cells overnight at 17°C. Cells were centrifuged and resuspended in 50 mM Tris-HCl buffer pH 8.0, containing 500 mM NaCl, 20 mM imidazole, and 5% glycerol. After cell lysis by sonication, the cells debris fraction was discarded and the supernatant, containing the N-terminally His-tagged protein was loaded on a chromatography column filled with HisTrap Ni^2+^ resin (GE Healthcare). Protein was eluted from the column using 400 mM imidazole. The 6xHis-tag was cleaved off by TEV protease during overnight dialysis at 4°C against 50 mM Tris-HCl buffer pH 8.0, containing 500 mM NaCl. The sample was again loaded on the Ni-affinity chromatography column and the tag-free flow-through was concentrated and loaded on a HiLoad 16/600 Superdex 200 (GE Healthcare) size exclusion (SEC) column. Protein was eluted using 25 mM Tris-HCl pH 8.0, containing 100 mM NaCl and 1 mM TCEP. The purity of the selected fractions was verified by SDS-PAGE, the fractions containing target proteins were pooled together. The protein samples were aliquoted and frozen at −80°C with 20% glycerol at a final concentration of ∼8 mg mL^-1^ until use. Prior to use, a protein sample was applied on the SEC column to remove the glycerol, concentrated, and then dialyzed overnight at 4°C against 30 mM carbonate buffer pH 9.0. Alternatively, the peak fraction after SEC purification was kept on ice for further testing, as it was observed that both enzymes at high concentration retain their activity without loss for a long period of time (up to 1 month) when kept on ice.

### 4.2 Generation of ReAIV K137A and ReAV K138A mutants

Site-directed mutagenesis of ReAIV and ReAV was carried out using the polymerase incomplete primer extension (PIPE) technique (Klock and Lesley, 2009). The lysine codons were changed to alanine at position 137 (ReAIV) or 138 (ReAV). The pET151D-*ReAIV* or pET151D-*ReAV* plasmids carrying the original protein sequences were used as templates for PCR amplification. To eliminate transformation background, template DNA was digested by DpnI restriction enzyme according to the manufacturer’s protocol. The reaction products were used for transformation of *E. coli* BL21 Gold (DE3) competent cells (Agilent Technologies). The mutations (underlined codons) were introduced using specific primers, as listed below:

ReAV-K138A_FWD: TGC​TCG​GGCGCGC​ACG​TCG​GCA​TGC​TTG​CC

ReAV-K138A_REV: CCG​ACG​TGCGCGCC​CGA​GCA​ATT​GCT​GCA​AAC​C.

ReAIV_K137A_FWD: TGT​AGC​GGTGCACAT​GCC​GGT​TTT​ATT​TGT​GCA​TGT​TGC.

ReAIV_K137A_REV: CCG​GCA​TGTGCACC​GCT​ACA​GTT​ATT​ATG​CAG​TGC​G.

All resulting vectors were verified by DNA sequencing and the mutated proteins were overexpressed and purified as described for the WT proteins.

### 4.3 The TPEN procedure

To remove bound Zn^2+^ ions, the purified protein was incubated for 2 h on ice with a 50-fold molar excess of TPEN (N,N,N′,N′-tetrakis (2-pyridylmethyl)ethylenediamine), dissolved in 96% ethanol. After incubation, the protein was concentrated and dialyzed for 2 h against 25 mM Tris-HCl buffer pH 8.0, containing 100 mM NaCl and 1 mM TCEP. Prior to ITC titrations, TPEN was removed by size exclusion chromatography on a HiLoad 16/600 Superdex 200 (GE Healthcare) column using the dialysis buffer.

### 4.4 Determination of K_D_ for zinc binding

Isothermal titration calorimetric (ITC) measurements of the interactions between ReAIV or ReAV and Zn^2+^ cations were conducted using an iTC-200 calorimeter (Malvern). The protein, after TPEN procedure (section 4.2), kept at the concentration of ∼110–200 µM in the sample cell, was titrated with 0.9–1 mM ZnCl_2_ (in the syringe). Both reactants were in the SEC buffer. The ZnCl_2_ titrant was injected in 2 µL aliquots until saturation. Raw ITC data were analyzed using PEAQ ITC software (Malvern) to obtain the thermodynamic parameters: stoichiometry (*N*), dissociation constant (K_D_), and changes in enthalpy (ΔH) and entropy. ‘One set of binding sites’ model was fitted to the data. Reference power was set to 5. Stirring speed of 750 rpm and time spacing of 150 s were used. Measurements were carried out in duplicate and averaged.

### 4.5 Nessler activity assay

L-Asparaginase activity under different conditions (described below) was determined by the Nessler method as described previously (Loch et al., 2023), with a slight modification. Briefly, the substrate (L-Asn) at 10 mM concentration and enzyme at a final concentration of ∼50 nM were incubated for 5 min at room temperature. After stopping the reaction with 1.5 M trichloroacetic acid (TCA), ultrapure water, a stabilizer solution (4 mM sodium tartrate with 10 mg mL^-1^ poly (vinyl alcohol), PVA) and Nessler reagent (Chempur) were added to the samples. The color reaction was quantified by absorption at 420 nm using a microplate reader (Hidex). The amount of ammonia liberated upon L-Asn hydrolysis was determined from a calibration curve prepared for (NH_4_)_2_SO_4_ concentration range of 0–6.5 mM.

To determine the effect of buffer systems and their concentration on the activity of ReAIV and ReAV, phosphate, Tris-HCl and carbonate buffer (pH 9.0) in the range 10–100 mM of ionic strength were applied.

Search for optimal Zn^2+^ ion concentration for the L-asparaginase activity of ReAIV and ReAV and their K137A and K138A mutants was conducted by pre-incubating the enzymes (at ∼50 nM concentration for both WT isoforms, and ∼0.11 µM and 1.5 µM for the K137A and K138A mutants, respectively) for 5 min with various ZnCl_2_ concentrations in the range of 1–100 µM. The enzymatic activity was evaluated in 20 mM Tris-HCl buffer (pH 9.0).

The assessment of NaCl impact on ReAIV and ReAV activity was performed in a 10 mM carbonate buffer, containing 1 or 2.5 µM ZnCl_2_, respectively, for ReAIV or ReAV. The enzyme (at 56 nM concentration) was preincubated for 5 min with various NaCl concentrations in the range 0.001–100 mM. The half-maximal inhibitory concentration (IC50) for Cl^−^ was calculated by fitting the obtained data with the dose-response inhibition model, with variable slope of the GraphPad Prism 6 software version 6.07.

The impact of several metal ions (Mg^2+^, Ca^2+^, Cd^2+^, Cu^2+^, Co^2+^, Hg^2+^, and Ni^2+^) on ReAIV and ReAV activity was tested by pre-incubating the enzyme (at final concentration ∼50–56 nM) with chloride salt of each divalent metal at 100 µM concentration. The reactions were carried out in 10 mM Hepes pH 8.5. For the strongest inhibitors, the IC50 values were determined in the presence of appropriate metal ions concentration. Measurements were made in triplicate. IC50 was calculated by fitting the data with the dose-response inhibition model, with variable slope of the GraphPad Prism 6 software version 6.07.

### 4.6 Determination of the kinetic parameters

The kinetic parameters of ReAIV and ReAV were determined by the multiple injection method using a MicroCal PEAQ-ITC (Malvern) microcalorimeter. First, the enthalpy of total conversion of all substrate into product (total molar enthalpy of the reaction, H_app_) was determined by injecting 2 µL of 10 mM L-Asn (Sigma-Aldrich) into the reaction cell containing 2 µM of the enzyme. Four injections were performed, separated by intervals long enough (150 s) to ensure total substrate conversion. After integration of each peak, the enthalpies of all injections appeared to be comparable and were averaged to obtain H_app_. Similar blank experiment was performed, in which 2 µL of 10 mM L-Asn was injected into the reaction cell containing buffer only. Since substrate dilution was an endothermic process, the absolute H_app_ value of the exothermic reaction was increased by the value of the heat from the blank experiment. Next, the differential power change (dQ/dt) arising from the turnover of the substrate into product was determined in a heat-rate shift experiment, in which the substrate at 100 mM concentration (in the syringe) was injected in nineteen 1.8 µL aliquots with short 60 s intervals (to minimize substrate depletion) into the reaction cell with the enzyme kept at 5–1 µM concentration, depending on sample activity at the given conditions (buffer) or for given variant (mutant). All measurements were taken at 37°C, with stirring at 700 rpm and differential power set to 10 μcal s^-1^. Raw rate data were analyzed using the Enzyme Kinetics–Multiple Injections fitting model implemented in the MicroCal PEAQ-ITC Analysis Software (Malvern). Briefly, they were transformed into reaction rates and L-asparagine concentrations and fitted with the Michaelis-Menten equation. The data point after the first injection was discarded as the heat of dilution of the substrate after the first injection was high, which could cause an error in reading the baseline level; subsequent injections resulted in much lower dilution heat. Final kinetic parameters were calculated by averaging the values obtained in two or three separate experiments. To track the impact of low micromolar Zn^2+^ concentration on the kinetic parameters, the kinetics were determined as described above in Tris-HCl buffer with the presence of optimal zinc concentration as determined before by the Nessler reaction (i.e., 1 μM for ReAV, 2.5 μM for ReAIV, and 5 or 10 μM for the K→A mutants of ReAIV or ReAV, respectively). To study the impact of the preselected inhibiting metal ions (Cu^2+^, Cd^2+^, Hg^2+^, Ni^2+^) on K_M_, the protein was aliquoted and flash frozen with 5% glycerol. The kinetics were determined in duplicate, in the presence of 2.5 μM of each of these metal ions in 8 mM carbonate buffer (except Hg^2+^, for which only 200 nM concentration was used, due to the low signal) and at 25°C. For K_M_ determined in the presence of each ion, K_M_/K_Mcontrol_ ratio (expressed as %) was calculated, and plotted (Figure 6C). To track the impact of the Cl^−^ anions, the kinetics parameters were determined in the presence of 20 mM NaCl in a 10 mM carbonate buffer. To study the impact of Zn^2+^, the kinetics were determined in the presence of optimal zinc concentration in 10 mM carbonate buffer (ReAIV, ReAV), or 10 mM (ReAIV and its K137A mutant) or 20 mM (ReAV and its K138A mutant) Tris-HCl buffer. To search the optimal temperature of ReAIV and ReAV activity, the kinetics were measured by ITC thermostated to the temperature of 10, 17, 25, 37, 45, 55, and 65°C in single experiments.

### 4.7 NanoDSF experiments

Thermal stability of the studied proteins was monitored by nanoDSF using a Prometheus NT.48 (NanoTemper Technologies) instrument. Melting scans were recorded by monitoring fluorescence emission at 330 and 350 nm for samples subjected to a 25°C–95°C temperature ramp at 1°C/min. ReAIV and ReAV at 18 μM concentration (in 20 mM Hepes 8.0) were incubated with an equimolar ratio of five metal ions: Zn^2+^, Cd^2+^, Hg^2+^, Cu^2+^, or Ni^2+^. The protein:metal molar ratio was 1:1. Additionally thermal stability was measured for both isoforms in buffer (50 mM Tris-HCl pH 8.0, 1 mM TCEP) with 10, 150 and 500 mM NaCl with or without equimolar ratio of Zn^2+^.

### 4.8 Mass photometry

Mass Photometry experiments were performed on a RefeynOne mass photometer (Refeyn, United Kingdom) provided by the Sample Preparation and Characterization (SPC) Facility, EMBL, Hamburg. All measurements were carried out using flow chambers assembled from microscope coverslip and silicone gasket. A blank buffer was loaded into the sample compartment and the objective was focused on the surface of the glass-buffer interface. Protein samples were diluted with the blank buffer, filtered through 0.22 µm filters, and then added into the buffer-containing well. The final concentration of the protein sample was in the 20–50 nM range. Data acquisition started within 10 s of sample addition for a total of 60 s, resulting in approximately 3000 particles detected in each acquisition. All buffers (50 mM Tris-HCl pH 8.0, 30 mM carbonate pH 9.0) and solutions (2.5 mM Zn^2+^, 2.5 mM Mg^2+^, 100 mM L-Asn) used were filtered through 0.22 µm filters. Images were processed using PhotoMol software and plotted as molecular mass distribution histograms.

### 4.9 Endotoxin level determination

Endotoxin level was determined using commercially available tests (Pierce Chromogenic Endotoxin Quantification Kit, Thermo Scientific) according to the instructions provided by the manufacturer.

## Data Availability

The data analyzed in this study is subject to the following licenses/restrictions: None restrictions apply to the dataset. Requests to access these datasets should be directed to sliwiak@ibch.poznan.pl.
