# Supplementary material for: Biochemical characterization of L-asparaginase isoforms from Rhizobium etli—the boosting effect of zinc
Source: Front Chem. 2024 Feb 22;12:1373312. doi: 10.3389/fchem.2024.1373312 (PMC10917881; doi:10.3389/fchem.2024.1373312)

Figure S1. Effect of 1 mM TCEP on the activity of ReAIV (A) and ReAV (B), determined by the Nessler method in 10 mM carbonate buffer pH 9.0, supplemented with 1 and 2.5 µM $ZnCl_2$, respectively. Bar graphs represent means from eight determinations, with their ±SD shown as error bars.

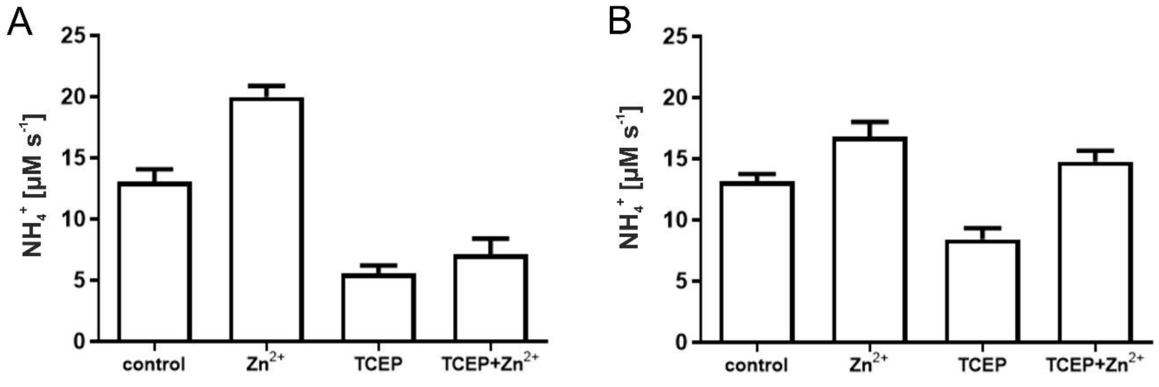

Figure S2. (A), (B) Raw data obtained after the first injections of ITC-MIM rate experiments as follows: 2 µL aliquots of different substrates at 100 mM concentration are injected into the cell containing 15 nM (in the case of the L-Asn substrate) or 1 µM of ReAIV (A) or ReAV (B) with other substrates.The right panels have zoomed insets of raw heat data obtained with substrates other than L-Asn. (C) H_{app} values of single injection of 14 µL of 100 mM acrylamide into the cell containing 1 µM of ReAIV, ReAV, BSA, or 10 mM carbonate buffer pH 9.0 only. (D) H_{app} experiment consisting of 4 subsequent 9 µL injections of 100 mM acrylamide into the cell containing 1µM of ReAIV the injections were separated with intervals long enough to enable total substrate conversion, and indicate strong product inhibition or an enzyme deactivation. (E) Raw data of the full ITC-MIM rate experiment, where 100 mM acrylamide is injected into the cell containing 1µM of ReAIV using 2 µL aliquots separated by short 60 second intervals. (F) Michaelis-Menten equation fitting to raw data obtained after first 6 injections of acrylamide, before visible product inhibition/enzyme deactivation.

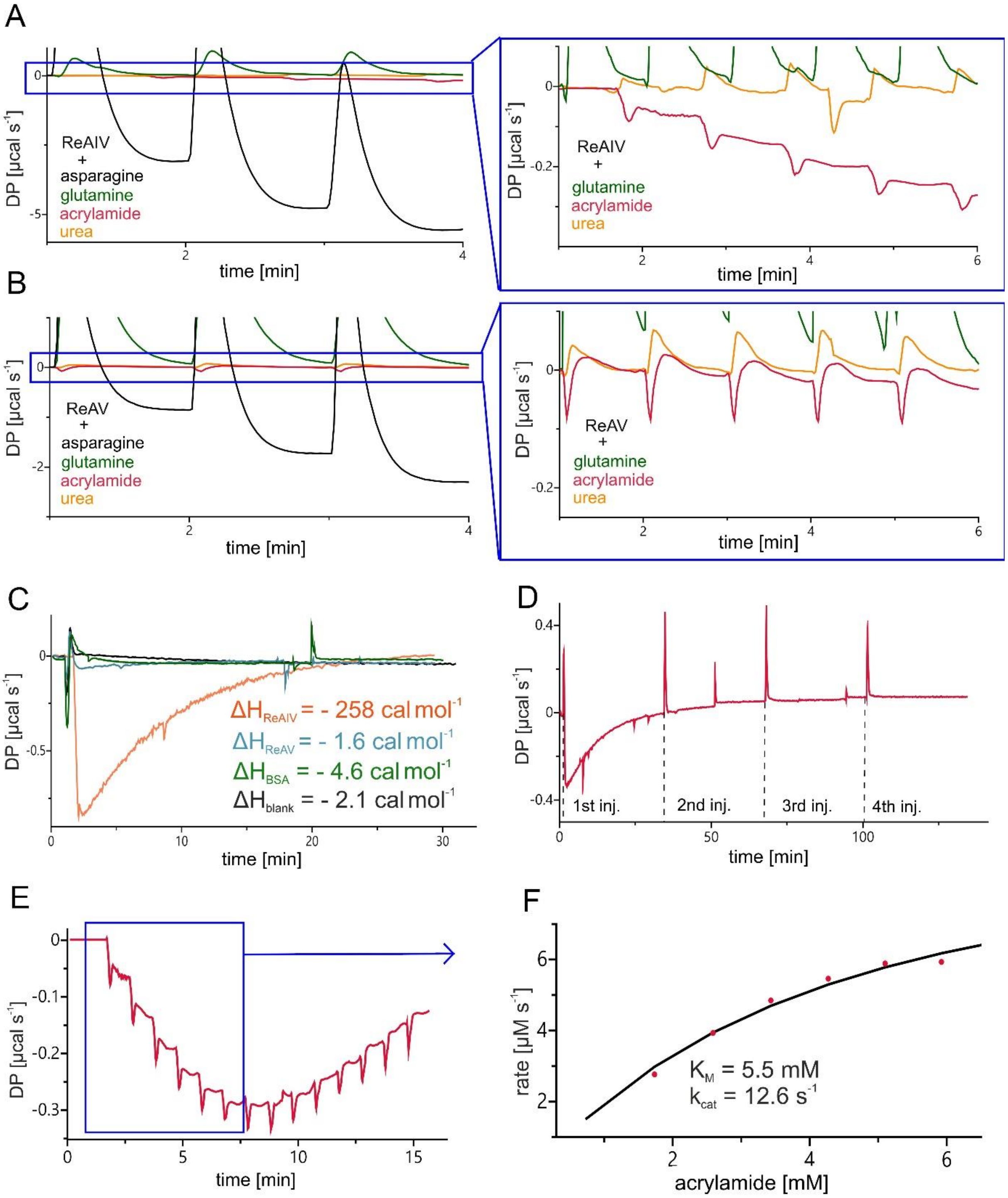
A
DP [µcal s-1]
ReAIV
+
asparagine
glutamine
acrylamide
urea
time [min]
B
ReAV
+
asparagine
glutamine
acrylamide
urea
C
ΔH_ReAIV = - 258 cal mol-1
ΔH_ReAV = - 1.6 cal mol-1
ΔH_BSA = - 4.6 cal mol-1
ΔH_blank = - 2.1 cal mol-1
D
1st inj.
2nd inj.
3rd inj.
4th inj.
E
F
rate [µM s-1]
K_M = 5.5 mM
k_cat = 12.6 s-1
acrylamide [mM]

Figure S3. Correlation between endotoxin levels and substrate specificity (K_M) of four ReAV preparations. The error bars indicate ±SD of the mean from two replicates.

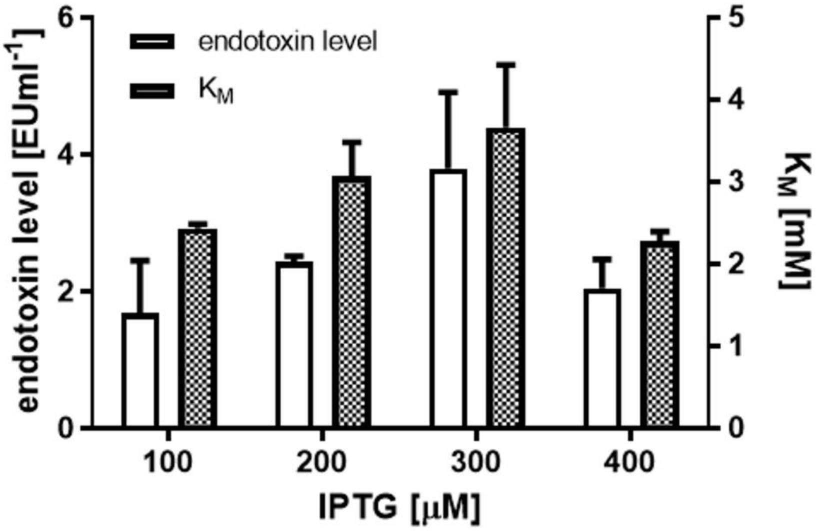

Supplement: Supplementary file 1 [file DataSheet1.pdf]
